# CNS Penetration of Intrathecal-Lumbar Idursulfase in the Monkey, Dog and Mouse: Implications for Neurological Outcomes of Lysosomal Storage Disorder

**DOI:** 10.1371/journal.pone.0030341

**Published:** 2012-01-18

**Authors:** Pericles Calias, Mikhail Papisov, Jing Pan, Nancy Savioli, Vasily Belov, Yan Huang, Jason Lotterhand, Mary Alessandrini, Nan Liu, Alan J. Fischman, Jan L. Powell, Michael W. Heartlein

**Affiliations:** 1 Shire Human Genetic Therapies, Inc., Lexington, Massachusetts, United States of America; 2 Department of Radiology, Massachusetts General Hospital, Boston, Massachusetts, United States of America; 3 Department of Radiology, Harvard Medical School, Cambridge, Massachusetts, United States of America; 4 Department of Nuclear Medicine, Shriners Burns Hospital, Boston, Massachusetts, United States of America; Biological Research Center of the Hungarian Academy of Sciences, Hungary

## Abstract

A major challenge for the treatment of many central nervous system (CNS) disorders is the lack of convenient and effective methods for delivering biological agents to the brain. Mucopolysaccharidosis II (Hunter syndrome) is a rare inherited lysosomal storage disorder resulting from a deficiency of iduronate-2-sulfatase (I2S). I2S is a large, highly glycosylated enzyme. Intravenous administration is not likely to be an effective therapy for disease-related neurological outcomes that require enzyme access to the brain cells, in particular neurons and oligodendrocytes. We demonstrate that intracerebroventricular and lumbar intrathecal administration of recombinant I2S in dogs and nonhuman primates resulted in widespread enzyme distribution in the brain parenchyma, including remarkable deposition in the lysosomes of both neurons and oligodendrocytes. Lumbar intrathecal administration also resulted in enzyme delivery to the spinal cord, whereas little enzyme was detected there after intraventricular administration. Mucopolysaccharidosis II model is available in mice. Lumbar administration of recombinant I2S to enzyme deficient animals reduced the storage of glycosaminoglycans in both superficial and deep brain tissues, with concurrent morphological improvements. The observed patterns of enzyme transport from cerebrospinal fluid to the CNS tissues and the resultant biological activity (a) warrant further investigation of intrathecal delivery of I2S via lumbar catheter as an experimental treatment for the neurological symptoms of Hunter syndrome and (b) may have broader implications for CNS treatment with biopharmaceuticals.

## Introduction

The brain is protected by the blood-brain barrier (BBB) [1], the blood-cerebrospinal fluid (CSF) barrier [2] and the avascular arachnoid epithelium [3]. Together, these barriers provide physical, transport and metabolic regulation by restricting the entry of macromolecules and polar solutes from the blood to the brain and spinal cord [1]. Most pharmacological agents are unable to penetrate the brain in sufficient amounts to have therapeutic benefits, with only highly lipophilic, small molecules (<500 Da) usually able to cross the BBB [4]. Thus, a major challenge for treatment of central nervous system (CNS) disorders, many of which are debilitating and life threatening, is the lack of convenient and effective methods for delivery of therapeutic agents to widespread regions of the brain.

Various noninvasive brain-targeting methods using endogenous molecular transport mechanisms have been explored as drug delivery strategies. These have included fusion proteins that target delivery by transcytosis using insulin [5], [6], or transferrin [7], [8], receptors. Encapsulation technologies, such as pegylated immunoliposomes, have been used to deliver plasmids to the brain through the BBB utilizing monoclonal antibody ligands as the targeting agent [9], [10]. More recent efforts have explored the use of nanoparticles (polymers, emulsions or suspensions) to traverse the BBB through various endocytotic pathways [11]. While promising from a mechanistic point of view, few of these innovative strategies have progressed beyond preclinical evaluation, leaving direct administration into the CSF or brain tissue as the only clinically viable method for delivery of therapeutics to the brain and spinal cord.

The two main routes of direct delivery to the CNS are intrathecal (IT) drug administration and direct intracerebroventricular (ICV) injection. IT drug administration is an established route for treatment of disorders such as chronic pain due to cancer or other conditions [12], [13], [14], and spasticity [13], [15]. Several IT drug delivery devices are marketed for these applications, with the benefits and inherent complications generally well understood [16].

ICV administration has also been used therapeutically, most notably for treatment of Parkinson's disease [17], [18], delivery of opioids for pain [19], and chemotherapy in children [20]. However, this administration route has not achieved the same level of use in clinical practice compared to IT drug delivery devices. While the disadvantages of ICV administration (invasiveness, need for specialized neurosurgical skills) are readily apparent, it is not clear whether benefits are to be expected when administering a drug, especially a large protein, into the ICV space versus the IT space at the midthoracic region. One possible advantage is that drugs delivered in the midthoracic region might be absorbed into the bloodstream or degraded locally before the CSF flow delivers them to the brain tissues whereas ICV-delivered drugs might have a better penetration rate into the brain parenchyma. Thus, it becomes a clinical imperative to compare the drug delivery and distribution patterns after IT versus ICV administration, in order to offer patients with devastating CNS diseases requiring protein therapy at the level of the brain a treatment modality with an optimal risk/benefit ratio.

As early as the 1960s, investigators reported that large molecular weight molecules may be distributed throughout the brain via the CSF following IT-lumbar delivery. Rieselbach et al [21] imaged the movement of radioactive colloidal gold (Au^198^) delivered into the lumbar sac of both monkeys and humans, showing widespread cerebral subarachnoid and ventricular system distribution of the particles primarily from CSF bulk flow. However, the distribution and tissue deposition of large molecules are complex, with variable CSF flow in different brain regions influencing the diffusion of molecules into surrounding tissues [22]. More recent investigations suggest that the mechanisms by which large molecules may be transferred from the CSF to brain parenchyma involve active transport mechanisms, including receptor mediated uptake, axonal transport and translocation along the Virchow-Robin channels [23], [24], [25].

Mucopolysaccharidosis (MPS) II (Hunter syndrome) is a rare inherited lysosomal storage disorder (LSD) primarily affecting children. The disease is caused by a deficiency of iduronate-2-sulfatase (I2S), with an estimated incidence of 1 per 162,000 live births [26]. Recombinant human I2S (idursulfase, Elaprase®; Shire Human Genetic Therapies, Inc., Cambridge, MA) is approved for treatment of certain somatic symptoms of Hunter syndrome but there is no pharmacological therapy for treatment of the neurological manifestations, which can include delayed development and progressive cognitive impairment [27]. I2S, a large, highly glycosylated enzyme (approximate molecular weight: 76 kDa) [28], does not traverse the BBB following intravenous (IV) administration [1]. However, upon direct administration to the brain parenchyma of animal models, the enzyme is readily absorbed by target cells via mannose-6-phosphate (M6P) receptors [29].

A clinical debate remains as to whether large molecular weight proteins can be delivered as effectively by the IT-lumbar route as by ICV administration. While studies in rodents suggest equivalency in brain tissue biodistribution of large molecules delivered via IT or ICV routes into the CSF [30], to our knowledge, this comparison has not been performed in large animal models or in humans. Consequently, ICV or intracisternal-IT routes both remain preferred methods of administration for delivery of recombinant proteins to deep brain tissues of animal models. Studies in the MPS I canine model, where recombinant iduronidase was administered by intracisternal injection, provide supportive evidence that enzyme delivered to the CSF may penetrate into deeper regions of the brain and modify disease pathology [31].

Here, we are the first to report results from a series of animal studies comparing the IT-lumbar and ICV delivery routes for a large molecule biologic, I2S. Direct comparisons were made using positron emission tomography (PET) in nonhuman primates and enzyme biodistribution in dogs. We observed similarly widespread distribution and cellular localization of I2S in the brain after both IT-lumbar and ICV delivery. In contrast, notably more I2S was detected in the spinal cord following IT delivery. Chronic CNS delivery of a specific I2S-IT formulation was effected via a SC port connected to an IT-lumbar catheter. This study revealed in greater detail the extent of enzyme biodistribution and cellular localization in the brain and spinal cord of nonhuman primates. Moreover, in a mouse model of MPS II, IT-delivered I2S produced morphological improvements in all areas of the brain evaluated.

Thus, contrary to the prevailing viewpoint that flow dynamics of the parenchyma interstitium [32] and CSF [33] would prevent penetration of IT-lumbar–administered proteins to the white matter of the brain, we clearly demonstrate that IT delivery of a lysosomal enzyme results in protein penetration to all brain tissues and deposition in the lysosomal compartment of target cells, the site of pathological glycosaminoglycan (GAG) accumulation. The less invasive nature of IT-lumbar delivery and the successful precedent of its usage in chronic pain and spasticity, make this route an attractive, clinically relevant means of delivering biological therapeutics to the brain, particularly in children.

## Results

### 1. PET Imaging of I2S Translocation in Cynomolgus Monkeys

**Figure 1:**
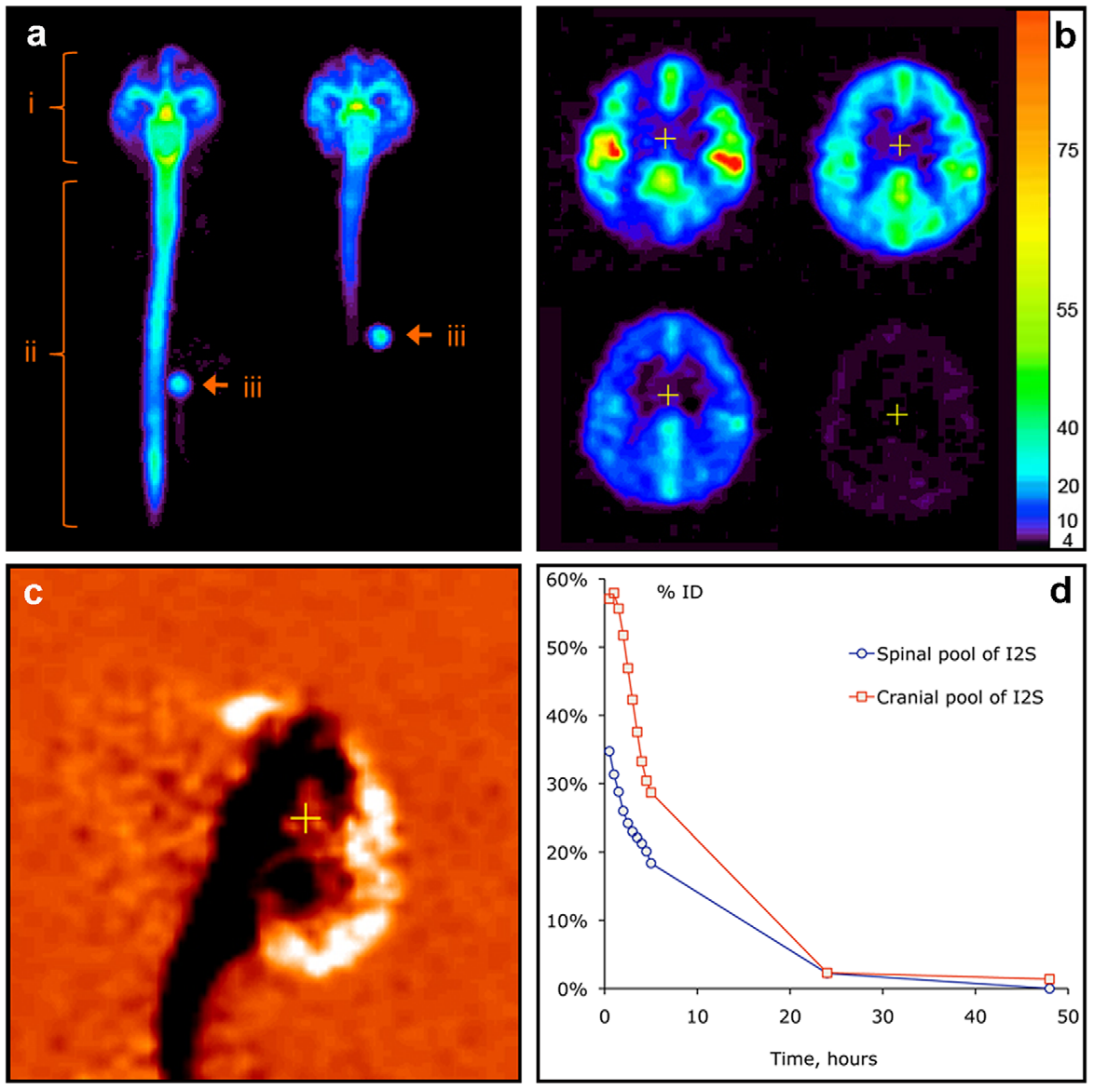
In vivo distribution of ^124^I -labeled I2S (3 mg/animal) in cynomolgus monkeys by PET. (**A**) Distribution of I2S administered through the lumbar (left) and ICV (right) catheters 30 minutes after the administration as demonstrated by a projection PET image (sum of all slices). Relative linear color scale. (**B**) The distribution of I2S in the brain at 0.5, 2.5, 5 and 24 hours after lumbar administration; PET image, 1.2 mm slice through the corpus callosum region in the plane parallel to the occipital bone. The color scale is calibrated in µg/ml of I2S. (**C**) Changes in the cerebral I2S distribution between 0.5 and 5 hours after lumbar administration shown in monochrome linear color scale. The image was obtained by subtraction of the quantitative data matrix obtained at 5 hours from the one obtained at 0.5 hours. Neutral orange color represents no change. Clearance of I2S from the CSF is seen as black, and accumulation in the parenchyma and arachnoid as white color. (**D**) An example of single-animal dynamics of I2S clearance from the leptomeningeal compartment and CNS.

Cynomolgus monkeys (*Macaca fascicularis*) were administered ^124^I -labeled I2S at three doses, 3 (*n* = 4), 10 (*n* = 1), and 20 (*n* = 1) mg by IT-lumbar injection and 3 mg (*n* = 1) by ICV injection; ^124^I-labeled I2S was also administered IV at 1 (*n* = 4) and 0.1 (*n* = 4) mg/kg. PET imaging data showed that ^124^I -labeled I2S administered through the IT-lumbar catheter spread immediately and uniformly in the CSF over 15–20 cm of the length of the spine, 10–15 cm cranially and 3–5 cm dorsally from the catheter tip (Figure 1A-D). Within 30 minutes after injection, I2S was distributed over the entire leptomeningeal space. While distribution of protein in the parenchyma was comparable for IT and ICV administrations, the ICV route resulted in notably less deposition within the spinal column (Figure 1A). By 1 hour, 55±20% of the dose was in the cranial region following IT-lumbar administration. Sequential PET imaging of the brain after the IT-lumbar injection (Figure 1B) demonstrated that I2S had moved from the CSF into the superficial (20–100 µg/ml as estimated from PET data) and then into deeper brain tissues (3–20 µg/ml). The inflow continued for 4±1 hours, as exemplified by the differential (0.5 to 5 hour) PET image in Figure 1C. In the cranial segments, the clearance was faster (Figure 1C), which is consistent with CSF drainage to the system predominantly in the arachnoid granulations of the superior longitudinal sinus [34]. No residual I2S deposition was detected near the catheter opening.

I2S transfer from CSF to the systemic circulation started immediately after the injection, suggesting significant uptake of drug from the lumbar CSF into the blood and distribution to the periphery. As no I2S deposition was observed in lymph nodes along the spine, the data are consistent with direct drainage of CSF into venous blood in the arachnoid as the major route of I2S clearance from the CNS. Approximately 40±10% of the dose was transferred to the systemic circulation within 5 hours, at which time I2S concentrations in the liver and heart were approximately 50% of the respective concentrations in the same organs resulting from IV injection at the same dose (Table 1). The kinetics of I2S accumulation outside the CNS were consistent with absorption from the CSF, with T_max_ of 4±1 hours and C_max_ of 0.1±0.5% injected dose/g for lung, heart and kidneys and 0.3±0.1% injected dose/g in the liver. I2S concentrations in the blood (measured by radioactivity at 5 hours postinjection) were similar to residual I2S concentrations in blood at the same time point after IV administration (12±5% of the initial concentration after IV administration). Therefore, these data suggest that the CSF serves as an intermediate reservoir for I2S, from which approximately half of the dose is gradually transferred into the systemic circulation over the first 4 hours after injection.

**Table 1:** Distribution of 124I-labeled I2S following positron emission tomography in cynomolgus monkeys at 5 hours after injection (normalized*).

Injection Route	Number of Animals	Total Dose	Gray matter (%ID/g)	Liver (%ID/g)	Heart (%ID/g)
IL	4	3 mg	0.77±0.43	0.14±0.02	0.09±0.02
IL	1	10 mg	0.242	0.24	0.11
IL	1	20 mg	0.465	0.15	0.08
IL	1	30 mg	0.479	0.14	0.10
IV	4	1 mg/kg**	0.025±0.004	0.37±0.10	0.13±0.07
IV	4	0.1 mg/kg**	0.058±0.053	0.44±0.14	0.14±0.02
ICV	1	3 mg	0.635	0.097	0.04

*Data normalized to 2 kg animal body weight.

**The total injected doses in the IV groups were 3.5±1 mg and 0.34±0.1 mg, respectively.

ICV, intracerebroventricular; ID, injected dose; IL, intralumbar; IV, intravenous.

Overall, data from PET analyses suggests that IT-lumbar administration is an efficacious delivery route for I2S to the CNS and, in parallel, to other organs. The results of enzyme delivery to the systemic circulation and other organs following IT-lumbar administration will be reported in detail elsewhere.

### 2. Cellular Localization of I2S in the CNS

Healthy cynomolgus monkeys received six consecutive monthly doses of I2S-IT (3, 30 and 100 mg/dose administered via an implanted IT drug delivery device with a SC port) as well as weekly IV I2S doses of 0.5 mg/kg. Device and vehicle controls (*n* = 6, all groups) were treated similarly with respect to dosing regimen. Both the IT device placement and the dosing regimen at the highest dose tested were well tolerated [35].

**Figure 2:**
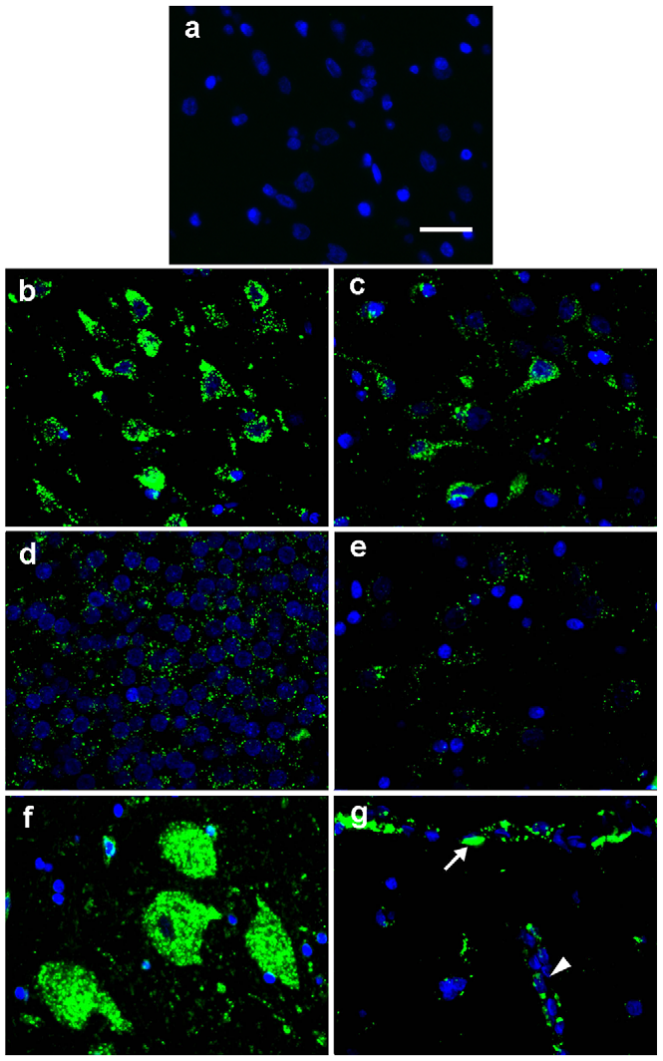
Cellular uptake of I2S in the neurons and vascular cells of the brain of monkeys. **(A)** I2S immunohistochemical staining of the cerebral cortex of vehicle control monkeys was negative. Representative images showing I2S following 6 monthly IT injections of 100 mg/dose detected in the neurons by immunohistochemistry in the cerebral cortex (**B**), thalamus (**C**), hippocampus (**D**), caudate nucleus (**E**) and spinal cord (**F**). (**G**) Meningeal cells (arrow) covering the surface of the cerebral cortex and perivascular cells surrounding the blood vessel (V) were also positive for I2S. Green, I2S; blue, 4′-6-diamidino-2-phenylindole (DAPI)-stained nuclei. Scale bar: 25 µm.

**Figure 3:**
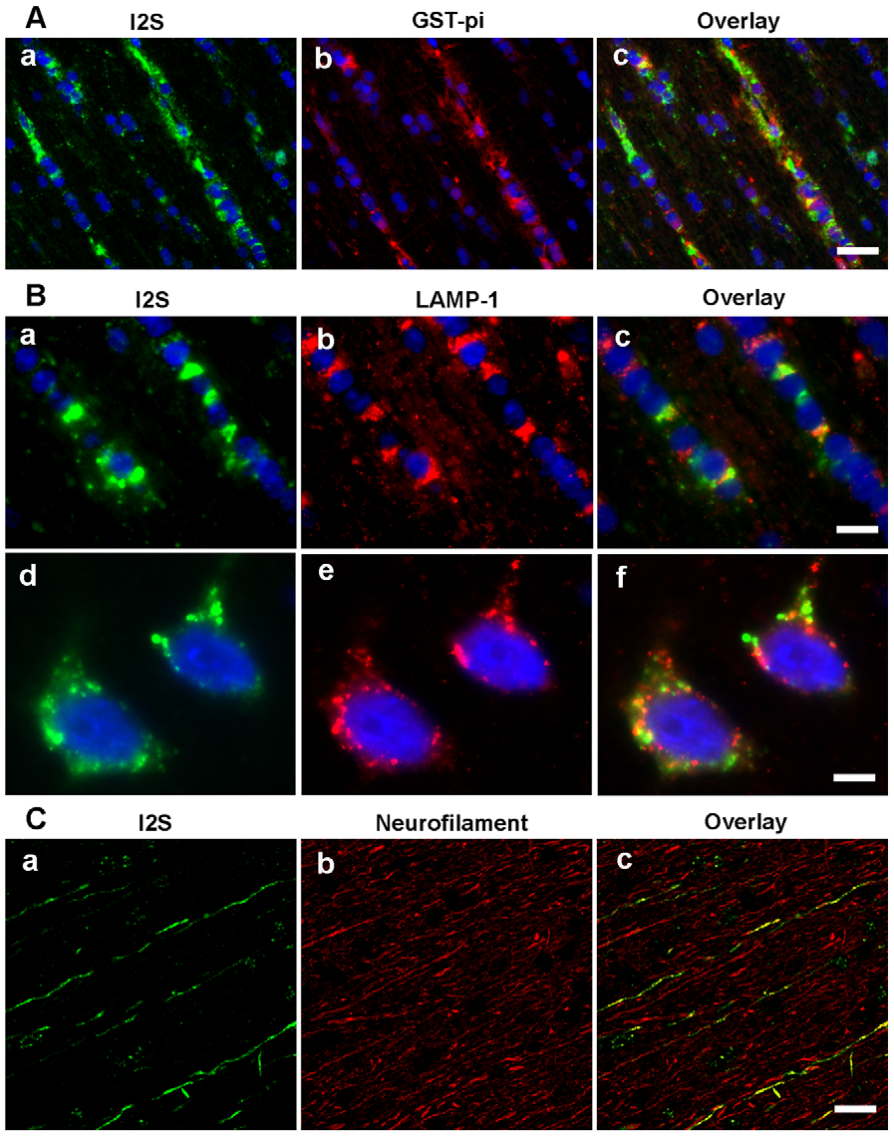
I2S is detected within the lysosomes of oligodendrocytes and in the axons of white matter. (**A**) Representative images showing I2S uptake in oligodendrocytes in the white matter of the 100 mg/dose IT-injected monkeys as demonstrated by colocalization of I2S (**a,** green) with glutathione-S-transferase-pi, an oligodendrocyte marker (**b,** red) and the overlay image (**c**). (**B**) I2S was located within lysosomes of the oligodendrocytes as demonstrated by colocalization of I2S (**a,** green) with lysosomal associated membrane protein-1 LAMP-1 (**b,** red) and the overlay image (**c**). I2S is located within the lysosomes of neurons, as demonstrated by colocalization of I2S (**d**, green) with LAMP-1 (**e**, red) and the overlay image (**f**). **(C)** I2S was also detected in some axons in the white matter as demonstrated by colocalization of I2S (**a**, green) with neurofilament, an axonal marker (**b,** red) and the overlay image (**c**). Scale bars: **A,** 25 µm; **B, a–c**, 10 µm, **d–f**, 5 µm; **C**, 30 µm.

Assessment of vehicle treated control animals revealed that the immunohistochemistry (IHC) procedure did not detect endogenous monkey iduronate-2-sulfatase and was specific for recombinant human I2S (Figure 2A). In contrast, there was widespread cellular deposition of I2S throughout the CNS in treated animals. In the gray matter, I2S was detected in neurons of the cerebrum, cerebellum, brainstem, and spinal cord of all groups in a dose-dependent manner. In the surface gray matter of the higher dose groups, large numbers of cerebral neurons were positive for I2S staining in the surface cortex (Figure 2B). I2S was also detected in neurons in the thalamus (Figure 2C), hippocampus (Figure 2D), caudate nucleus (Figure 2E) and spinal cord (Figure 2F). Meningeal and perivascular cells also stained positive for I2S (Figure 2G). These data are consistent with the results from our PET studies described above. While the I2S staining density in the white matter was generally lower than that in the gray matter, I2S was detected within oligodendrocytes (Figure 3A). As shown in Figure 3B, I2S was located within the lysosomes of oligodendrocytes and detected specifically within the lysosomes of neurons. I2S was also detected within axons (Figure 3C).

In order to discern whether the delivered I2S retained biological activity, levels of I2S in the brain were measured utilizing a specific activity assay. The enzyme activity in the brain of the 3 mg IT dose group 24 hours after the last dose was not apparently different from the basal levels in the device- and vehicle-control animals. Enzyme activity in the brain of 30 mg and 100 mg IT-dosed animals was above baseline at necropsy (24 hours postdose). Results of the quantitative analysis of I2S in brain tissues from this study are described in detail elsewhere [35].

**Figure 4:**
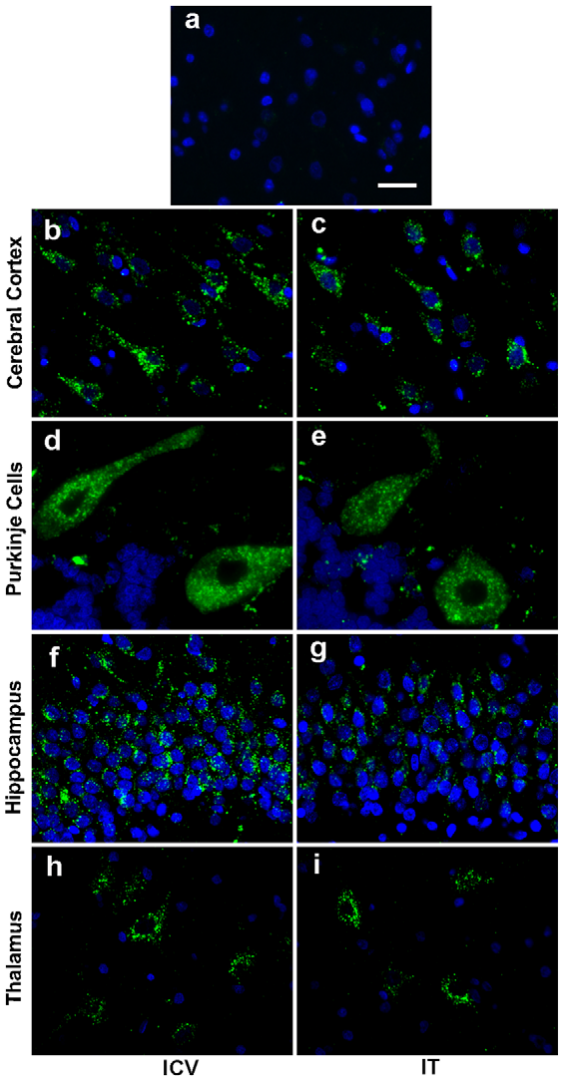
Widespread distribution in the brains of dogs following either intracerebroventricular (ICV) or intrathecal (IT) administration. **(A)** I2S immunohistochemical staining of the cerebral cortex of vehicle control dogs was negative. Representative fluorescent immunohistochemical images showed uptake of I2S in the neurons of both ICV-dosed (**C,D,F,H**) and IT-dosed (**C,E,G,I**) dogs. I2S was detected in neurons in the deep internal layer of the cerebral cortex (**B,C**), Purkinje cells of the cerebellum (**D**,**E**) and neurons in the hippocampus (**F,G**) and thalamus (**H,I**). Green, I2S; blue, DAPI-stained nuclei. Scale bar: 25 µm.

The observed I2S distribution pattern was analogous to healthy beagle dogs given a single IT or ICV dose. No endogenous dog iduronate-2-sulfatase was detected in vehicle treated control animals by IHC confirming the specificity of the immunostaining procedure for recombinant human I2S (Figure 4A). I2S was widely distributed throughout the gray matter of both IT and ICV groups as determined by IHC. In the cerebral cortex, neurons were positive for I2S in all six neuronal layers, from the surface molecular layer to the deep internal layer in both IT and ICV groups (Figure 4B and 4C). In the cerebellar cortex of the IT and ICV groups, I2S was detected in neurons, including Purkinje cells (Figure 4D and 4E). In both IT and ICV groups, a large population of neurons in the hippocampus was positive for I2S (Figure 4F and 4G). I2S-positive neurons were also found in the thalamus and caudate nucleus in both groups (Figure 4H and 4I).

### 3. IT-Lumbar–Administered I2S Improves Brain Pathology in an MPS II Model

**Figure 5:**
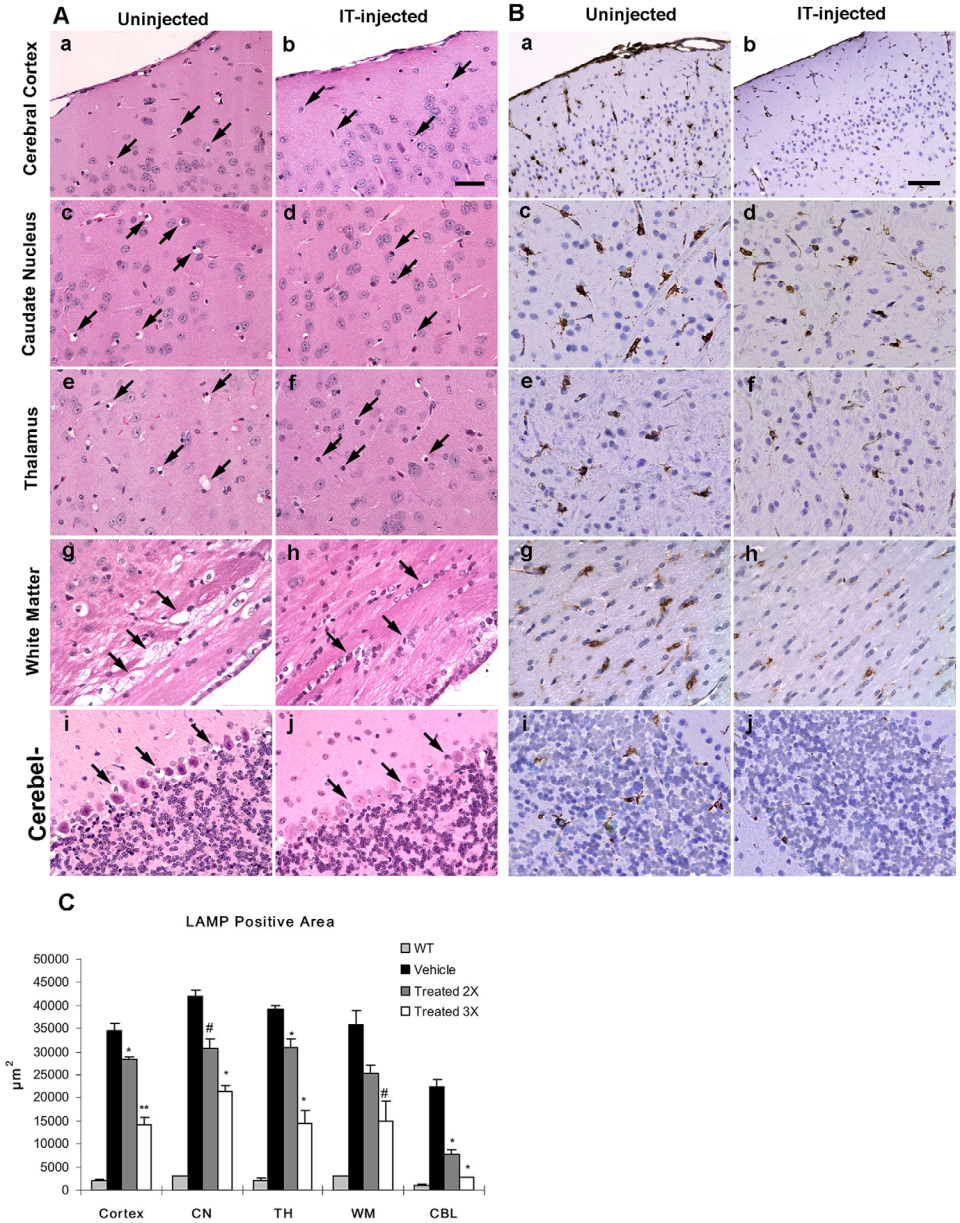
Reversal of pathology in I2S knockout (mucopolysaccharidosis II) mice after three IT-lumbar injections of I2S. (**A**) Hematoxylin and eosin-stained brain tissues of uninjected (left panels) and injected (right panels) mice showed numerous cellular storage vacuoles (arrows) in the uninjected brain that were markedly reduced in injected mice in the cerebral cortex (**a,b**), caudate nucleus (**c,d**), thalamus (**e,f**), white matter (**g,h**), and cerebellum (**i,j**). Scale bar: 25 µm. (**B**) As demonstrated by immunohistochemical staining of lysosomal-associated membrane-1 (LAMP-1), there was a marked reduction of LAMP-1 immunoreactivity in the brains after three IT injections of I2S (right panels) compared with uninjected mice (left panels). There was a decrease in the number of LAMP-1 positive cells and lighter staining intensity in the cerebral cortex (**a,b**), caudate nucleus (**c,d**), thalamus (**e,f**), white matter (**g,h**), and cerebellum (**i,j**). Scale bar: 25 µm. **(C)** A comparison of the mean LAMP-1 positive area between uninjected and I2S (two or three IT injections) injected wild-type (WT) mice in the cerebral cortex (cortex), caudate nucleus (CP), thalamus (TH), white matter (WM) and cerebellum (CBL). Data are represented as the mean ± s.d. # P<0.05; * P<0.01; ** P<0.001.

**Figure 6:**
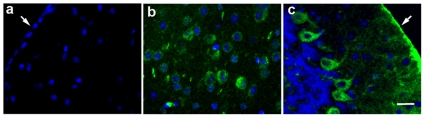
Cellular uptake of I2S in IT-injected (three injections) I2S knockout mice. (**A**) I2S immunohistochemical staining of the cerebral cortex of untreated control mice was negative. In IT-injected mice, I2S positive staining was found in neurons of the cerebral cortex (**B**) and Purkinje cells of the cerebellum (**C**). Meningeal cells (arrows) were also I2S positive in IT-injected animals. Scale bar: 25 µm.

**Figure 7:**
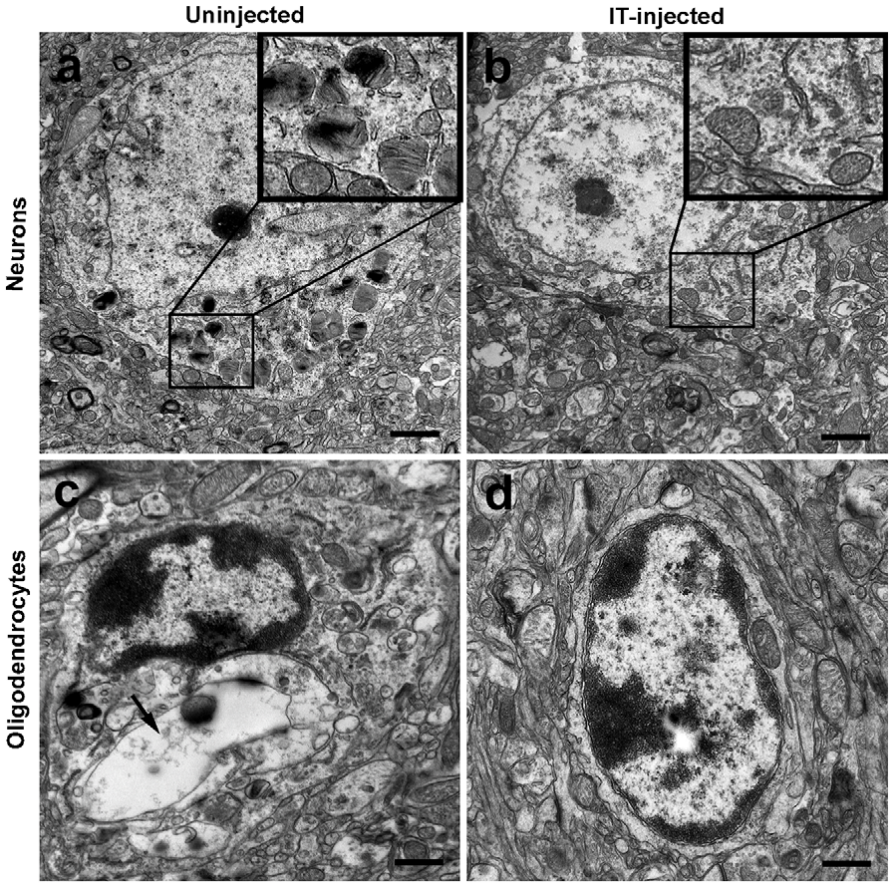
Electron micrographs of brain cells in uninjected and IT-injected (3 doses) I2S knockout mice. Pathological improvements occurred at the ultrastructural level. (**A**) Neurons of uninjected mice had lamellated inclusions, zebra body-like structures and vacuoles containing granular storage material (insert), which was reduced in I2S injected mice (**B**). Oligodendrocytes of uninjected mice showed large electron-lucent storage vacuoles (arrow; **C**) while oligodendrocytes of I2S-injected mice had minimal vacuolation (**D**).

Currently, the only animal model of MPS II disease is an I2S gene knockout generated in mice [36]. I2S was administered to 8-12 week old male mice via direct lumbar injection (260 µg; two injections at study days 1 and 8 or three injections at study days 1, 8 and 15). Mice were sacrificed 1 hour after the final injection followed by tissue preparation for IHC and histopathological examinations. Following the third injection, there was widespread reduction of cellular vacuolation in the surface cerebral cortex, caudate nucleus, thalamus, cerebellum, and the white matter in I2S-treated mice compared to control (untreated) mice (Figure 5A, a-j). The IT-treated mice also had marked reductions in lysosomal-associated membrane protein-1 (LAMP-1) immunoreactivity, a lysosomal protein marker used for the detection of lysosomal storage disorders and an indicator of disease state, in the surface cerebral cortex, caudate nucleus, thalamus, cerebellum and white matter (Figure 5B, a-j). Morphometrical analysis of LAMP-1 immunostaining of various brain regions confirmed that there were significant reductions in the LAMP-1 positive immunostaining in all areas of the brain evaluated (cortex, caudate nucleus, thalamus, cerebellum and white matter; Figure 5C). As demonstrated by IHC, I2S was not detected in uninjected MPS II control animals (Figure 6A), but was detected in the neurons of the cerebral and cerebellar cortex and the meningeal cells (Figure 6B and 6C) of treated animals. Additionally, electron microscopy showed there was a reduction in the presence of storage inclusions in neurons in the gray matter and vacuolation in oligodendrocytes in the white matter (Figure 7A-D).

## Discussion

While much progress has been made in treating the somatic symptoms of MPS disorders [37], [38], development of a therapy for the devastating neurological involvement is greatly needed. In severe Hunter syndrome, histological changes in the brains of affected patients include atrophy, cortical neuronal swelling, cerebral white matter reduction, dilated perivascular spaces and Purkinje cell dendrite swelling [39]. Magnetic resonance imaging/spectroscopy studies have shown that severe diffuse lesions involving the white matter, brain atrophy and hydrocephalus were more common in patients with cognitive impairment than in those without impairment [40]. However, even patients without cognitive impairment or developmental delays were shown to have brain abnormalities that included atrophy, ventriculomegaly and enlarged perivascular spaces [41].

Several strategies have been evaluated to correct the neurological aspects of LSDs but these have either been unsuccessful or are still in preclinical development. Bone marrow transplants (BMT) or hematopoietic stem cell transplants (HSCT) appear to have limited or no success in treating the neurological and cognitive impairment associated with Hunter syndrome. Guffon et al [42] found improvements in somatic symptoms in children with Hunter syndrome who were treated with BMT, with two patients with the attenuated phenotype having normal social and scholastic development; four patients with the severe syndrome had postgraft declines in intelligence/developmental quotients and three lost the ability to walk in their early teens. Limitations to BMT/HSCT may be that only a small number of transplanted cells migrate into the CNS and differentiate into microglia, and the time required for the number of cells to be transplanted may be too long to prevent disease progression [43].

Viral vectors, primarily adeno-associated virus (AAV) [44], [45] and lentiviruses [46], [47], [48] expressing recombinant enzymes have also been evaluated, generally in mouse models, for virus-mediated enzyme replacement therapy in the brain [44], [45], [46], [47]. Cross-correction (in which corrected cells release enzyme into extracellular space that is taken up by receptor-mediated endocytosis on neighboring cells) and enzyme transport along neural projections are reported to be the primary mechanisms responsible for widespread brain distribution [45]. Secretion of enzyme from transduced cells into the CSF may also contribute to the distribution of enzyme replacement therapy [48]. However, one limitation to the use of viral vectors is that direct injection into the brain parenchyma is required [49]. It is estimated that as many as 368 injections would be needed to distribute AAV vectors to the infant brain [50]. Another limitation is that lentivirus vectors can integrate into the genome of dividing and nondividing cells [51]–[52]. Since lentivirus vectors strongly prefer transcriptional units for integration [51], [53], there is the potential for inactivation of essential genes or activation of proto-oncogenes. Although postmitotic cells such as neurons are unlikely to be affected, neural stem cells in the brain may be susceptible [49]. AAV vectors also integrate into active genes, with frequent chromosomal deletions of up to 2 kB at integration sites [54].

The current studies are the first to demonstrate that direct CNS administration of a recombinant lysosomal protein results in the delivery of a significant fraction of the administered protein to the brain and widespread deposition in neurons of the brain and spinal cord in both cynomolgus monkeys and dogs. The similarities observed in brain distribution patterns achieved after IT-lumbar and ICV administration of I2S are suggestive of bulk flow and active remixing of the CSF. Thus in a clinical setting, both administration routes might be effective in delivering drug to target cells in the CNS. Spinal injection ports have two benefits over ICV injection: (1) they are perceived as less invasive and (2) they have been used successfully for many years in the treatment of chronic pain and spasticity, the latter indication being especially relevant for the pediatric population. Additionally, the observed deposition of I2S in the spinal cord following IT administration indicates that the IT-lumbar route may provide a third advantage over ICV administration in addressing spinal components of complex CNS disorders.

Evidence from perivascular cell staining and protein translocation dynamics observed by PET imaging demonstrates that the enzyme moves within the perivascular space, presumably by pulsation-assisted convective mechanisms. An additional mechanism of transport is suggested by the observed association of I2S with neurofilaments, indicative of active axonal transport. The latter presumably begins with protein interaction with neuronal M6P receptors [55], which are widely expressed on cells of the spinal cord and brain [56]. Axonal transport of lysosomal enzymes has been implied by indirect methods in vivo [44] and by imaging in vitro [57]. The current study provides the first direct evidence of axonal transport of nonvirally mediated enzyme replacement delivered via the CSF. Thus, protein delivery from the CSF to the brain surface and deeper into the brain seems to depend on active transfer processes, none of which have been previously described for protein delivery to the brain. Further elucidation of these transport mechanisms and their dependence on protein structure will establish the general applicability of this approach for treatment of other CNS disorders.

We have demonstrated that IT-lumbar administration of I2S results in cellular deposition of the enzyme in the brain and spinal cord of cynomolgus monkeys and dogs, and associates with the lysosomes. Moreover, in a knockout mouse model of MPS II, I2S treatment by IT-lumbar administration led to a decrease in cellular vacuolation, a hallmark of the pathology associated with this disease. Overall, these data demonstrate the physiological transport of a therapeutic protein from CSF to the brain parenchyma after IT-lumbar administration, thus suggesting a clinically feasible route for delivery of enzyme replacement therapeutics to the brain. Studies in humans utilizing this approach are currently under way.

## Materials and Methods

### Ethics Statement

The studies involving animals were performed at Association for Assessment and Accreditation of Laboratory Animal Care (AAALAC)–accredited facilities and were performed in accordance with the Guide for the Care and Use of Laboratory Animals (7^th^ Edition, 1996, National Research Council, U.S.). The following details regarding the welfare of non human primates and steps taken to ameliorate suffering are provided in accordance with the recommendation of the Weatherall report, “The use of non-human primates in research”. Non human primates were housed under temperature, humidity, and lighting controlled conditions in accordance with recommendations in the Guide for the Care and Use of Laboratory Animals. For example, rooms were set to maintain 22±2°C, 50% ±30% relative humidity, 12 hour light:dark cycle, and with 10 room air changes per hour. Appropriate food, water, treats and vitamin supplements were provided and animals were given access to environmental enrichment such as approved toys, swings, perches, mirrors, television, or music to promote psychological well-being. Every effort was made to minimize pain, discomfort and suffering through the use of appropriate methods and agents for analgesia, anesthesia and euthanasia as described below. All animals were under the care and supervision of a veterinarian.

The 6-month cynomolgus monkey and Beagle dog studies were performed at Northern Biomedical Research, Inc. (Muskegon, MI) in accordance with the Guide for the Care and Use of Laboratory Animals, United States Department of Health and Human Services, No. 86-23, and the Animal Welfare Act (9 CFR Part 3); USDA No. 34-R-0025. The studies were approved by the Northern Biomedical Research, Inc. Institutional Animal Care and Use Committee (IACUC) (Cynomolgus monkey study protocol number 047-004; Beagle study protocol number 047-001). Non-invasive PET imaging in cynomolgus monkeys was performed at Massachusetts General Hospital (Boston, MA) in accordance with the Guide for the Care and Use of Laboratory Animals and the Animal Welfare Act; USDA No. 14-R-0014. The study was approved by the Massachusetts General Hospital IACUC (Protocol number 2003N00128). The studies in mice were performed at Shire Human Genetic Therapies (Lexington, MA) and were approved by the Shire Human Genetic Therapies IACUC (Protocol number ACUP 47).

### 1. Iduronate-2-sulfatase (I2S)

Recombinant human I2S was expressed and purified from a human-derived cell line [58] and was provided in IT (idursulfase-IT) [35], and IV (commercially available idursulfase) formulations for the non-human primate and Beagle studies. For the mouse studies, I2S was concentrated and suspended in phosphate buffered saline (PBS).

### 2. Positron Emission Tomography Study in Cynomolgus Monkeys

Male and female cynomolgus monkeys (*Macaca fascicularis*) between 1.8–5.6 kg were administered ^124^I-labeled I2S at three doses, 3 mg (6 mg/ml; *n* = 4), 10 mg (20 mg/ml; *n* = 1) and 20 mg (40 mg/ml; n = 1), by IT-lumbar injection and 3 mg (6 mg/ml; *n* = 1) by ICV injection. In addition,^124^I-labeled I2S was administered IV at two doses (1 mg/kg, *n* = 4; 0.1 mg/kg, *n* = 4; see Table 1). Over the entire duration of the study, the monkeys were segregated from other nonhuman primates and housed in a separate room at the Massachusetts General Hospital primate facility. Animals were fasted for 24 hours before each experiment. At the housing site, the animals were sedated with ketamine hydrochloride IM combined with xylazine (IM; Rompun, Bayer AG; Leverkusen, Germany) and then transported to the imaging site. The animals were intubated and given continuous Isoflurane (Halocarbon Products Corp., River Edge, NJ)/oxygen anesthesia. Heart rate, breathing rate and carbon dioxide content in the exhaled air were monitored continuously; isoflurane flow was adjusted as needed. Animals were given nonradioactive iodine solution (0.2 ml, 15 mM sodium iodide) SC immediately before the study to suppress ^124^I uptake in the thyroid. The radioiodinated proteins were administered IV or IT.

The study used a microPET P4 scanner (Siemens/CTI Concorde Microsystems, Knoxville, TN) designed for small primates (opening 22 cm, axial field of view [FOV] 7.89 cm, transaxial FOV 19 cm). The energy window of the detector for the entire study was set to 350–650 keV and coincidence timing window was set to 6 nanoseconds. We have determined previously that the imaging data under these settings are fully quantitative and linear at ^124^I doses of up to 1 mCi per animal. ^124^I was supplied as sodium ^124^I solution in 0.02 M sodium hydroxide, 0.3–2.7 µL/MBq (IBA Molecular, Richmond, VA). The nominal radiochemical purity was 95% (<5% of iodate and diiodate by high-performance liquid chromatography [HPLC]) and the nominal radionuclide purity was >99% at calibration (<0.5% of ^123^I; ^125^I, none detected by high-purity germanium gamma spectroscopy). The chemical purity was Te<1 µg/ml by ultraviolet (UV)-visible spectroscopy.

I2S was labeled with ^124^I, up to 185 MBq/mg, using Iodogen (Pierce Biotechnology, Inc., Rockford, IL) precoated iodination tubes (pH, 7.4; 25°C) for 20 minutes, with subsequent metabisulfite treatment desalting and purification of the iodinated protein by size exclusion HPLC (SEC HPLC). Typically, the reaction mixture consisted of 0.5 M sodium phosphate buffer solution, 0.05 ml; protein (6–50 mg/ml), 0.05 ml; and [^124^I] sodium iodide, 0.02–0.07 ml (1.5–3 mCi). After iodination, the reaction mixture was transferred to a polypropylene tube containing 1.0±0.2 mg of dry sodium metabisulfite to deactivate the oxidants present in the aqueous phase. The solution was then centrifuged at 5 rpm for 1 minute and desalted on Sephadex G-25 (GE Healthcare, Milwaukee, WI) equilibrated with unbuffered isotonic saline. The iodinated protein was analyzed by SEC HPLC using a Bio-Sil SEC 125 column (BioRad Laboratories, Inc., Hercules, CA), 80×7.8 mm, with UV (wavelength 280 nm) and gamma radioactivity detection. The elution buffer was 20 mM sodium citrate, pH = 6.5, in 0.9% sodium chloride solution. The amount of protein was determined on the basis of the UV absorption at 280 nm; yield by protein was found to be nearly 100%. Radiochemical yield (by ^124^I) was 85±7% and the radiochemical purity was >98%. In a separate experiment, we have determined that iodination under these conditions did not affect the rate of protein uptake by cells or the subsequent intracellular activity as determined in vitro in cell-based assays (data not shown).

The radiolabeled preparation (containing 0.5–0.7 mCi of ^124^I per animal) was mixed with a calculated amount of unlabeled I2S to obtain the desirable protein dose. The solution was distributed into 0.5 or 1 ml syringes. The total administered volume was 0.4–1 ml, depending on the protein dose.

For IV administration, a catheter equipped with a T connector was inserted in the saphenous vein nonsurgically. The animals were set on a microPET bed and a whole body transmission image was acquired before the injection. The animals then were positioned for dynamic imaging of the lower thoracic (heart and liver) area. The protein solution was administered through the T connector cap and flushed with 1 ml saline simultaneously with the start of the dynamic imaging data acquisition. The latter was carried out for 20 minutes. Static whole body images were acquired (section by section) continuously for 5 hours and then at 24 and 48 hours. Blood samples were taken with each imaging session and were studied by HPLC with gamma detection.

For CNS administration, I2S was administered through SC injection ports equipped with catheters surgically implanted into the upper lumbar segment of the leptomeningeal space or the left ventricle for IT-lumbar and ICV delivery, respectively. A needle equipped with a T connector was inserted into the port. The animals were set on a microPET bed and a whole body transmission image was acquired before the injection. The animals were positioned for dynamic imaging of the area of port opening into the cerebrospinal fluid. The I2S solution was administered through the T connector cap and flushed with saline (1 ml + 0.5 ml per kg of body weight) simultaneously with the start of the dynamic imaging data acquisition. The dynamic acquisition was carried out for 20 minutes. Whole body images were acquired (section by section) continuously for 5 hours and then at 24 and 48 hours. Blood samples were taken at 40 minutes and 2 hours and studied by HPLC with gamma detection.

Data acquisition, prereconstruction and reconstruction were carried out on a Dell Precision PWS690 Workstation (Dell, Inc., Round Lake, TX; 3 GB RAM and 8 Xeon 3.20 GHz processors running under a 64-bit Windows XP [Microsoft Corp., Redmond, WA]) using Siemens MicroPET Firmware/Software, release 2.4 (Siemens Medical Solutions, Inc., Malvern, PA). All subsequent image processing and analyses were performed on nonhost workstations using the ASIProVM software (Siemens/CTI Concorde Microsystems, Knoxville, TN) under Windows XP and MacOS 10.5 (Apple Computer, Inc., Cupertino, CA) and AMIDE [59] running under MacOS 10.5. The data were reconstructed into the image matrix with the pixel size of 0.95 mm and fixed slice thickness of 1.2 mm using a 3-dimensional (3-D) ordered-subset expectation maximization/maximum *a posteriori* (OSEM3D/MAP) protocol [60], [61] with the hyperparameter *β* value of 1.516. The data were also reconstructed with Fourier rebinning 2-D filtered backprojection (FORE-2DFBP) to ensure that the numerical data derived from OSEM3D/MAP and FORE-2DFBP reconstructed images were identical and thus excluded possible reconstruction artifacts (none were identified). FORE-2DFBP was performed with a ramp filter cutoff at the Nyquist spatial sampling frequency (0.5 mm^−1^) [62]. Whole body images were composed of the acquired section images with a 12 mm overlap.

The images were processed to obtain numerical data from hand-drawn 3-D regions covering all organs and tissues of interest. Typically, each region of interest was 0.2–5 ml in volume. The data (expressed in nCi per ml) were converted into protein concentration and percent injected dose/ml, and tabulated. For the spine, the data were also obtained for each 1.2 mm slice of the spinal column and expressed as protein amount per spinal column length (µg/mm). Data were processed to evaluate the statistics (mean values ± standard deviations). Corrections typical for PET data evaluation (radionuclide decay, scatter, randoms, etc.) were made as in our previous studies [63].

### 3. Six-month Cynomolgus Monkey Study

Forty-eight male cynomolgus monkeys were randomized into one of five groups. For the experimental design of the study, see Table 2. All had catheters implanted in the subarachnoid space at the lumbar spine that terminated in a SC titanium access port. Prednisolone sodium succinate (intravenous [IV], 30 mg/kg) and flunixin meglumine (intramuscular [IM], 2 mg/kg) were administered prior to the surgical procedure and monkeys were pretreated with SC atropine sulfate (0.04 mg/kg), sedated with ketamine hydrochloride (IM, 8 mg/kg; Ketalar, Pfizer, Inc. New York, NY), intubated and maintained on approximately1 L/minute of oxygen and 2% isoflurane. An incision was made over the dorsal processes of the lumbar spine (L_4_, L_5_ or L_6_), and a hemilaminectomy was made for the insertion of a tapered polyurethane catheter (58.4 cm in length with six side holes of 0.33 mm diameter) at L_3_, L_4_ or L_5_. The catheter was inserted through a small dural incision and was advanced approximately 10 cm anterograde to the area of the thoracolumbar junction. A titanium SC port was attached to the IT catheter and implanted in the SC tissue. Proper catheter placement was confirmed by myelogram using Isovue-300 (0.8 ml; Bracco Diagnostics, Inc., Princeton, NJ). After recovering from surgery, animals received butorphanol tartrate (IM, 0.05 mg/kg) and ceftiofur sodium (IM, 5 mg/kg twice daily [BID] for 2 d). IV infusions were initiated at least 6 days after device implantation, with IT doses administered 2 days before every fourth weekly IV dose.

**Table 2:** Design of I2S biodistribution study in cynomolgus monkeys.

Group	Number of animals	IV Dose (mg/kg)*	IT Dose (mg)*	Last Day on Study (number of animals)	
				6 Months	Recovery
1	6	DC (NS)	DC (PBS)	6	-
2	12	0 (vehicle)	0 (IT vehicle)	6	6
3	12	0.5	3	6	6
4	6	0.5	30	6	-
5	12	0.5	100	6	6

*I2S unless otherwise specified.

DC, device control; IT, intrathecal; IV, intravenous; NS, normal saline; PBS, phosphate-buffered saline, pH 7.2.

The I2S-IT formulation was supplied in a vehicle of 154 mM sodium chloride and 0.005% polysorbate 20 (pH 6.0) at concentrations of 3, 30 and 100 mg/ml. Six monthly doses of I2S-IT were administered as a 1 ml bolus followed by a flush of 0.5 ml phosphate buffered saline (PBS). Commercially available I2S was used for IV administration (weekly doses of 0.5 mg/ml). Monkeys in the vehicle-control group received I2S-IT vehicle and I2S-IV vehicle. Device control animals received PBS (pH 7.2) for IT administration and 0.9% sodium chloride for IV administration. Clinical signs were monitored. Monkeys were sacrificed 24 hours after the final dose or following a 1-month recovery period. For a detailed description regarding the harvesting of tissues for histopathology, IHC, and quantitative I2S analysis, please see Felice et al [35].

#### 3.1. Determination of I2S activity in tissue extracts

I2S activity was determined with a 2-step fluorometric assay [64] using the substrate 4-methylumbelliferyl-α-iduronate-2-sulfate (Moscerdam Substrates, Rotterdam, the Netherlands). Tissue extracts were diluted in 0.2% bovine serum albumin (pH and heat-treated to inactivate lysosomal enzymes) supplemented with 0.004% sodium azide. Substrate was desulfated by duplicate incubations of 10 µl of diluted sample with 20 µl of 1.25 mM substrate for 4 hours at 37°C. After I2S inhibition by the addition of 20 µl of phosphate/citrate buffer (0.2 M Na_2_HPO_4_/0.1 M citric acid, 0.02% sodium azide, pH 4.7), samples were incubated with 10 µl of lysosomal enzymes purified from bovine testis for 24 hours at 37°C to liberate 4-methylumbelliferyl from substrate desulfated in the first reaction. This second reaction was stopped by adding 200 µl of stop buffer (0.5 M NaHCO_3_/Na_2_CO_3_, pH 10.7 with 0.025% Triton X-100) to each well. Fluorescence was measured in 96-well fluorometry plates using a SpectraMax M2 fluorescent plate reader (Molecular Devices, Sunnyvale, CA). Results were calibrated using 4-methylumbelliferone (MP Biomedicals) as a standard and were normalized to total protein in tissue extracts as determined by bicinchoninic acid assay. I2S activity was expressed as nmol of substrate hydrolyzed per hour per mg of total protein (nmol/h/mg protein).

### 4. Beagle Dog Study

Male beagle dogs were randomized using computer-generated numbers into two groups (group 1 [ICV], *n* = 3; group 2 [IT]; *n* = 4). All had catheters implanted in the subarachnoid space at the lumbar spine or in the left lateral cerebral ventricle (for dosing) and in the cisterna magna (for sampling). All catheters terminated in a SC titanium access port. Dogs were pretreated with atropine sulfate (SC, 0.04 mg/kg) followed by sodium thiopental (IV, 16 mg/kg) and were masked to a surgical plane of anesthesia, intubated and maintained on 2% halothane or isoflurane. Prednisolone sodium succinate (IV, 30 mg/kg) and flunixin meglumine (IM, 2 mg/kg), were administered prior to surgery.

To place the catheter in the cisterna magna, a longitudinal incision was made from approximately C_4_ to the occipital crest and the musculature was reflected. A hemilaminectomy (approximately 5 mm) was performed in the posterior portion of C_1_. An incision was made in the dura, and the catheter (0.9 mm outside diameter [OD] x 0.5 mm inside diameter [ID] stepped polyurethane catheter with polished stainless steel tip) was directed toward the cisterna and secured in place using dental acrylic. To place the catheter in the IT lumbar space, an incision was made over the dorsal process of the lumbar spine at approximately L_4_, L_5_ or L_6_. The muscle was dissected, and a hemilaminectomy was performed for the insertion of a 0.9 mm OD x 0.5 mm ID stepped polyurethane, fenestrated catheter. The catheter was advanced to the area of the thoracolumbar junction. Proper catheter placement was confirmed by myelogram with Isovue-300. For group 1 dogs, the insertion was at L_5_ with the catheter tip located at L_1_. For group 2 dogs, the insertion was at L_3_, L_4_ or L_5_ with the catheter tip located at L_1_ or T_12_. The skin was closed with sutures and tissue adhesive.

Magnetic resonance imaging (MRI) was performed to determine the coordinates for ICV catheter placement in the group 1 dogs. A dorsal sagittal incision was made over the calvarium, and a 0.9 mm OD x 0.5 mm ID polyurethane, fenestrated catheter was inserted in the left lateral cerebral ventricle through a craniotomy using stereotaxic techniques and anchored with dental acrylic. Dogs received postsurgical MRIs while under anesthesia to verify catheter placement; because one dog died during the postsurgical scan due to prolonged anesthesia, subsequent scans were not conducted.

Upon recovery from anesthesia, the dogs received butorphanol tartrate (IM, 0.05 mg/kg), for analgesia and ceftiofur sodium (IM, 5 mg/kg BID; one injection during or prior to surgery followed by three injections). The iduronate-2-sulfatase (I2S) infusions were started a minimum of 2 days after implantation of the delivery devices. An additional dog was used as an undosed surgical control. A single bolus 1 ml injection of I2S (30 mg/ml in 20 mM sodium phosphate, pH 6.0; 137 mM sodium chloride; 0.02% polysorbate-20) was administered IT or ICV followed by a 0.3 ml flush with PBS, pH 7.2. Clinical signs were monitored. Sacrifice occurred 24 hours postdose.

#### 4.1. Tissue processing for IHC in the dog study

Six 3 mm formalin-fixed brain slices (numbers 1, 4, 7, 10, 13 and 16) from each dog were numbered 1–6 (rostral to caudal). Brain slices 1–4 were from the levels of the forebrain, caudate putamen and thalamus/hypothalamus of the cerebrum, respectively. The posterior two slices contained the midbrain, cerebellum and brain stem (medulla oblongata) tissues. Cervical, thoracic, lumbar spinal cord and liver samples were also collected. At the time of tissue trimming, brain slice 1 was separated into left and right hemispheres and processed in two separate cassettes. Brain slices 2–6 were cut into quadrants due to their larger size. Each section was separated into quadrants (left and right hemispheres with upper and lower sections) and processed in separate cassettes. Transverse sections of each spinal cord sample were processed separately.

### 5. Mouse MPS II Model

The I2S knockout mouse model of MPS II was developed using a targeted disruption of the I2S locus that results in an accumulation of GAG in tissues and organs [65]. Six groups of male I2S knockout mice, 8–12 weeks old, were either treated with I2S (10 µl; 26 mg/ml) or left untreated. Groups A and B (*n* = 3) were administered three doses (at study days 1, 8 and 15) and two doses (at study days 1 and 8) of I2S, respectively. Group C and E (*n* = 3) were untreated control groups and group F (*n* = 3) was an untreated wild-type control. Group D was also treated with three doses at study days 1, 8 and 15. Animals were sacrificed 1 hour after the final dose of I2S. For the study, a concentrated I2S solution was prepared by dialyzing I2S against four changes of 2 L phosphate-buffered saline (PBS) followed by centrifugation using a Vivaspin column (Sigma-Aldrich, St. Louis, MO). The solution was resuspended in 1 ml PBS for a concentration of 51 mg/ml, which was subsequently diluted to the final dosing concentration. For the injection procedure, mice were anesthetized with 1.25% 2,2,2 tribromoethanol (intraperitoneally, 240–350 mg/kg) and the skin was prepped with povidone iodine followed by isopropyl alcohol. A small (1–2 cm) midline incision was made over the lumbosacral spine, and I2S (10 µl) was injected using a 32-gauge needle with a GASTIGHT® 10–20 µl glass Hamilton syringe (Hamilton Medical, Inc., Reno, NV) at a rate of 2 µl/20 seconds. The skin was closed with wound clips. Sacrifice was performed 1 hour after the final injection. Before tissue collection, mice were perfused with saline followed by 10% neutral buffered saline (groups A, B and C) or 4% paraformaldehyde (groups D, E and F). Histology, IHC and electron microscopy were performed on brain tissues. The area of LAMP-1–positive cells was analyzed with Image-Pro Plus software (Media Cybernetics, Inc., Bethesda, MD), and comparative statistics were performed using the Student's *t* -test.

### 6. Histological and Immunohistochemical Staining Procedures

Tissues were embedded in paraffin and serial sections were cut and stained with hematoxylin and eosin for histological evaluation. For IHC analysis in the dog and monkey studies, deparaffinized slides were incubated overnight with mouse monoclonal antibody 2C4-2B2 (Maine Biotechnology Services, Portland, ME) as the primary antibody to detect injected I2S (or mouse IgG as a control antibody; Vector Laboratories, Burlingame, CA). Following an overnight incubation at 2-8°C, a secondary goat anti-mouse IgG conjugated with horseradish peroxidase was added (Promega). After additional 30 minutes of incubation at 37°C, Tyramide-Alexa Fluor 488 labeling solution (Invitrogen Corp., Carlsbad, CA) was added for an additional 10 minutes. Slides were cover slipped using an antifading mounting medium (VectaShield; Vector Laboratories) containing 1.5 µg/ml 4′-6-diamidino-2-phenylindole as a nuclear counterstain and observed with a multiple channel Nikon fluorescent microscope. Representative digital images were captured for documentation.

Additional primary antibodies used for IHC included rabbit anti-glutathione-S-transferase-pi (Assay Designs, Inc., Ann Arbor, MI) for the detection of oligodendrocytes, rabbit anti-lysosomal-associated membrane protein-1 (anti-LAMP-1; Santa Cruz Biotechnology, Inc., Santa Cruz, CA) for the detection of LAMP-1 and rabbit anti-neurofilament 200 (Sigma-Aldrich) for the detection of axons. Rabbit IgG was used as a control primary antibody (Vector Laboratories). The secondary antibody was goat anti-rabbit Alexa Fluor 568 (Invitrogen).

For the mouse studies, 5 µm paraffin sections were prepared for hematoxylin and eosin I2S IHC staining. The fluorescence procedure was similar to that used above for the dog and monkey studies. For the LAMP-1 staining procedure, deparaffinized slides were incubated overnight with rat anti-LAMP-1 IgG (Santa Cruz Biotechnology) as the primary antibody or rat IgG2a as a control antibody (AbD Serotec, Raleigh, NC). Following overnight of incubation at 2–8°C, biotinylated rabbit anti-rat IgG (H&L) mouse adsorbed (Vector Laboratories) was added. Following 30 minutes of incubation at 37°C, samples were washed and then treated with avidin-biotin-peroxidase complex (Vector Laboratories) for 30 minutes. Labeled protein was localized by incubation with 3,3′-diaminobenzidine.
